# Overexpressed LAPTM4B-35 is a risk factor for cancer recurrence and poor prognosis in non-small-cell lung cancer

**DOI:** 10.18632/oncotarget.10907

**Published:** 2016-07-28

**Authors:** Fanming Kong, Fangfang Gao, Jun Chen, Yiyu Sun, Ying Zhang, Honggen Liu, Xiaojiang Li, PeiYing Yang, Rongxiu Zheng, Geli Liu, Yingjie Jia

**Affiliations:** ^1^ Department of Oncology, First Teaching Hospital of Tianjin University of Traditional Chinese Medicine, Tianjin, China; ^2^ Department of pediatrics, Tianjin Medical University General Hospital, Tianjin, China

**Keywords:** lung cancer, non-small-cell lung cancer, LAPTM4B-35, cancer recurrence, prognosis

## Abstract

**Background:**

The expression levels and clinical significances of Lysosomal-associated protein transmembrane-4β-35 (LAPTM4B-35) protein are unknown in the non-small-cell lung cancer (NSCLC). This study aimed to explore the expression and prognostic value of LAPTM4B-35 in NSCLC patients.

**Methods:**

The clinicopathological and survival data of 107 NSCLC patients who received radical surgery from 2007 and 2011 were reviewed. The LAPTM4B-35 expression of the paired tumors and adjacent normal specimens were detected, and the association between LAPTM4B-35 and clinical variables was explored. Kaplan-Meier analysis and Cox regression (Proportional hazard model) were performed to investigate the prognostic significance for NSCLC.

**Results:**

LAPTM4B-35 was over expressed in NSCLC tissues. The elevated LAPTM4B-35 expression was associated with cancer recurrence (*P* = 0.031). The 5-year median OS and PFS were significantly worse in the LAPTM4B-35 overexpressed group. Multivariate Cox analysis showed that LAPTM4B-35 over-expression was an independent factor for OS and PFS in NSCLC(*P* = 0.018, *P* = 0.026, respectively).

**Conclusions:**

The *o*verexpressed LAPTM4B-35 was an independent prognostic biomarker for NSCLC, which could predict cancer recurrence and poor over survival. And that may be applied as potential target for NSCLC treatment.

## INTRODUCTION

Lung cancer was the most important cause of tumor-related death, though the incidence began declining in the last decades [1-3]. As a subgroup of lung cancer, about 85% of all cases were the Non-small cell lung cancer (NSCLC) [4]. Despite advances in the diagnosis and treatment for the NSCLC, the 5-year survival rates for advanced NSCLC remains poor (less than 15%) [5, 6]. Compared to clinical characteristics, more novel and prognostic biomarkers that could predict the tumor recurrence and prognosis is still urgent to be identified to improve the prognosis of NSCLC [7].

Lysosome-associated protein transmembrane-4 beta (LAPTM4B) was first identified from hepatocellular carcinoma (HCC), and the LAPTM4B-35 overexpression was associated with poor prognosis of HCC [8]. And the similar results were also confirmed in various malignant tumors, such as pancreatic cancer [9], ovarian cancer [10], gallbladder carcinoma [11], and cervical cancer [12]. Recently, one study reported that LAPTM4B was elevated in Small cell lung cancer (SCLC) and its overexpression was an independent factor in SCLC prognosis [13]. However, the expression level and the association between LAPTM4B-35 and survival in NSCLC have not been rigorously and systematically evaluated.

In the current study, we investigated the LAPTM4B-35 expression levels and its association with clinical variables; also we further explored and discussed the prognostic value of LAPTM4B-35 in NSCLC. Our data showed that LAPTM4B-35 overexpression might be used as an predictor for NSCLC.

## RESULTS

### Patient clinicopathological characteristics

Table 1 summarized the clinicopathological characteristics of all the NSLCL patients. Among these patients, the median age in this study was 65 years (range, 28 to 81 years), and 85 (79.4%) patients were male and 22(20.6%) patients were female. 34(31.8%) patients were SCC and 73(68.2%) were non-SCC. 48 lymph node negative patients received postoperative adjuvant chemotherapy alone, while 55 lymph node metastasis patients had chemo-radiotherapy treatment.

### LAPTM4B-35 was overexpressed in NSCLC cancer tissues

**Figure 1:**
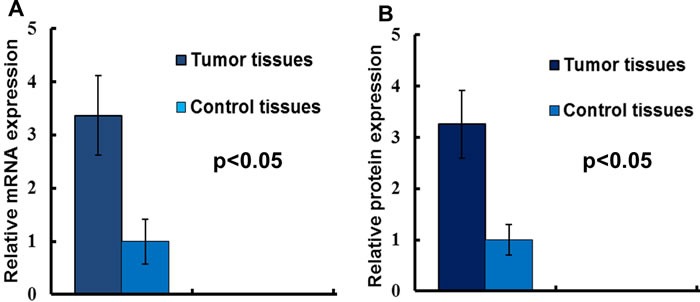
The LAPTM4B-35 expression in NSCLC **A.** LAPTM4B-35 mRNA expressions in NSCLC tissues and adjacent normal tissues. The results of the real-time qRT-PCR were analyzed by using the 2^-ΔΔCt^method. **B.** The relative protein expression.

The mRNA level of LAPTM4B-35 was detected by qRT-PCR, and as the Figure 1A indicated, the LAPTM4B-35 mRNA was overexpressed in tumor tissues. Consistently, our Western Blotting data showed the LAPTM4B-35 protein expression was also significantly elevated in cancer tissues (Figure 1B).

### LAPTM4B-35 expression and clinicopathological variables

We further compared the clinicopathological parameters based on the LAPTM4B-35 protein level. As the Table 1 showed, the elevated LAPTM4B-35 protein expression was significantly correlated with histology (*p* = 0.022) and clinical stage (*p* = 0.001). In detailed, the patients with overexpressed LAPTM4B-35 had poorly differentiation and advanced clinical stage. What's more, our data indicated that the increased LAPTM4B-35 protein level correlated with tumor recurrence (*p* = 0.031).

**Table 1:** Clinicopathological characteristics with LAPTM4B protein expression in NSCLC(*n* = 107)

Variables	Number	LAPTM4B protein expression		*P* value
		Low (*n*=53)	High (*n*=54)	
Age (years)				
≥65	56(52.3%)	29 (54.7%)	27 (50.0%)	0.264
<65	51(47.7%)	24 (45.3%)	27 (50.0%)	
Gender (%)				
Male	85(79.4%)	41(77.4%)	44(81.5%)	0.338
Female	22(20.6%)	12(22.6%)	10 (18.5%)	
Performance status				
ECOG 0-1	98(91.6%)	49 (92.5%)	49 (90.7%)	0.540
ECOG 2	9 (8.7%)	4(7.5%)	5 (9.3%)	
Pathological type				
SCC	34(31.8%)	18 (34.0%)	16 (29.6%)	0.235
Non-SCC	73(68.2%)	35 (66.0%)	38 (70.4%)	
Primary tumor size (cm)				
<5cm	66(61.7%)	32 (60.4%)	34 (63.0%)	0.187
≥5cm	41(38.3%)	21 (39.6%)	20 (37.0%)	
Histology				
Well differentiated	20(18.7%)	11(20.8%)	9(16.6%)	0.022
Moderately differentiated	41(38.3%)	30(56.6%)	11(20.4%)	
Poorly differentiated	46(43.0%)	12(22.6%)	34(63.0%)	
Tumor location				
Left	51(47.7%)	26 (49.1%)	25 (46.2%)	0.576
Right	56(52.3%)	27 (50.9%)	29 (53.8%)	
Lymph node metastasis				
No	59(55.1%)	27 (50.9%)	32 (59.3%)	0.083
Yes	48(44.9%)	26 (49.1%)	22 (40.7%)	
Clinical stage				
I	34(31.8%)	20 (37.8%)	14 (26.0%)	0.001
II	52(48.6%)	28 (52.8%)	24 (44.4%)	
III	21(19.6%)	5 (9.4%)	16 (29.6%)	
Thoracic irradiation				
No	46(43.0%)	23 (43.4%)	23 (42.6%)	0.474
Yes	61(57.0%)	30 (56.6%)	31 (57.4%)	
Tumor recurrence				
No	66(61.7%)	42(79.2%)	24 (44.4%)	0.031
Yes	41(38.3%)	11(20.8%)	30 (55.6%)	

SCC squamous cell carcinoma; Non-SCC(non-squamous cell carcinoma) includes adenocarcinoma, adenosquamous carcinoma, anaplastic large-cell carcinoma, sarcoma, adenoid cystic carcinoma, mucoepidermoid carcinoma and carcinoid tumor.

### LAPTM4B-35 protein expression and survival

**Figure 2:**
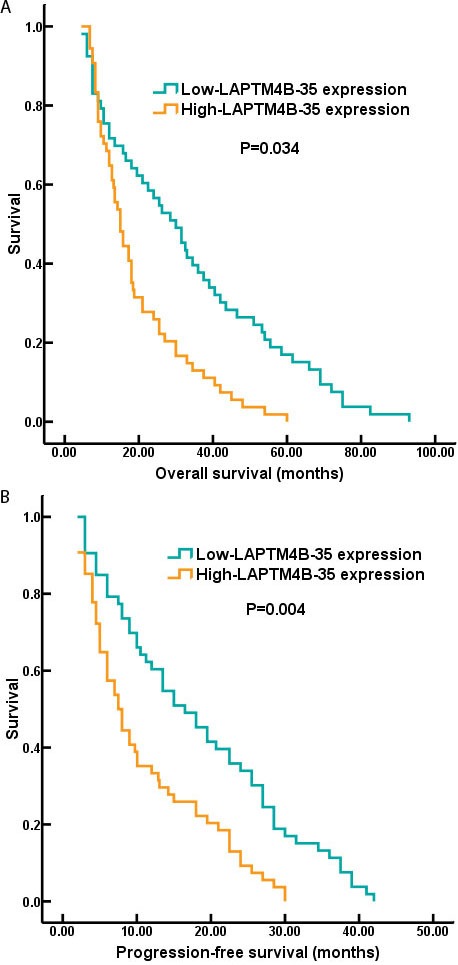
The survival curves of NSCLC patients with LAPTM4B-35 protein expression **A.** The 5 year median OS was shorter in patients with high-LAPTM4B-35 protein expression compared to those patients with low-LAPTM4B-35 protein expression (*p* = 0.034). **B.** The 5 year median PFS was statistically shorter in the high LAPTM4B-35 protein expression group than low LAPTM4B-35 protein expression group (*p* = 0.004).

Kaplan-Meier analysis showed that high LAPTM4B-35 protein expression was associated with poor prognosis in NSCLC patients. In detailed, the 5 year median OS was shorter in LAPTM4B-35 protein over-expression group (*p* = 0.034; Figure 2A). As the Figure 2B showed, the 5 year median PFS was statistically shorter in the high LAPTM4B-35 protein expression group than low LAPTM4B-35 protein expression group (*p* = 0.004).

Cox proportional hazards model was performed to identify if LAPTM4B-35 protein expression was an independent prognostic factor for NSCLC. After the univariate analysis, histopathologic differentiation (*p* = 0.055), the clinical stage (*p* = 0.028) and LAPTM4B-35 protein expression (*P* = 0.004) were associated with PFS (Table 2), while the histopathologic differentiation (*p* = 0.016), Lymph node metastasis (*p* = 0.042), the clinical stage (*p* = 0.010) and LAPTM4B-35 protein expression (*P* = 0.034) were associated with OS (Table 3). The multivariate analysis confirmed that histopathologic differentiation (HR = 1.681, 95% CI, 1.241-3.552; *p* = 0.039), the clinical stage (HR = 1.877, 95% CI, 1.346-6.043; *p* = 0.040) and elevated LAPTM4B-35 protein level (HR = 2.750, 95% CI, 1.911-4.607; *p* = 0.026) were independent factors for PFS (Table 2). While the clinical stage (HR = 2.924, 95% CI, 1.988-5.697; *p* = 0.001) and the elevated LAPTM4B-35 protein level (HR = 2.879, 95% CI, 1.621-4.318; *p* = 0.018) (Table 3) were identified as independent factors for OS.

**Table 2:** Univariate and multivariate analyses for PFS (*n* = 107)

Variable	Univariate analysis	Multivariate Cox regression		
	*p*-Value	HR	95% CI	*p*-Value
Age (years)	0.545	-	-	-
Gender	0.379	-	-	-
Primary tumor size	0.071	-		-
Histology	0.020	1.681	1.241–3.552	0.039
Primary tumor location	0.401	-	-	-
Lymph node metastasis	0.055	-	-	-
Clinical stage	0.028	1.877	1.346-6.043	0.040
LAPTM4B-35 expression	0.004	2.750	1.911-4.607	0.026

Progression-free survival (months)

**Table 3:** Univariate and multivariate analyses for OS

Variable	Univariate analysis	Multivariate Cox regression		
	*p*-Value	HR	95% CI	*p-*Value
Age (years)	0.247	-	-	-
Gender	0.842	-	-	-
Primary tumor size	0.089	-		-
Histology	0.016	1.250	1.101–3.157	0.336
Primary tumor location	0.332	-	-	-
Lymph node metastasis	0.042	1.819	0.997-3.001	0.877
Clinical stage	0.010	2.924	1.988-5.697	0.001
LAPTM4B-35 expression	0.034	2.879	1.621-4.318	0.018

## DISCUSSION

In the present study, we first explored the LAPTM4B-35 expression in NSCLC, and then investigated the association of LAPTM4B-35 protein expression with clinicopathological factors and prognosis. Our study found that the LAPTM4B-35 protein was expressed in NSCLC, and the elevated LAPTM4B-35 protein expression was associated with poor histopathologic differentiation (*p* = 0.022), advanced clinical stage (*p* = 0.001) and cancer recurrence (*p* = 0.031). The LAPTM4B-35 protein expression was further identified as independent prognostic factors for OS and PFS (*P* = 0.018, *P* = 0.026, respectively).

Lung cancer remained the most common cause of cancer related death worldwide. There are about 200,000 new lung cancer patients will be diagnosed in the new year [1]. NSCLC accounted for about 85% of all lung carcinomas, and it included non-SCC and SCC [6, 16]. Systematic clinical studies and basic research on NSCLC had improved the prognosis of the NSCLC, however, the long-term outcome of the NSCLC remained poor [17-20]. It is significant to identify the new biomarkers to improve the prognosis of NSCLC.

LAPTM4B was originally identified in hepatocellular carcinoma. And further studies found LAPTM4B-35 was overexpressed in several kinds of solid tumors [21, 22]. What was more, LAPTM4B-35 played critical role in tumorigenesis and tumor metastasis [23, 24]. Recently, Qiao and her colleagues reported that the LAPTM4B-35 expression was up regulated in SCLC patients [13]. Unfortunately, there was not too much information about the expression and clinical significance of LAPTM4B-35 in NSCLC. Therefore, we detected the LAPTM4B-35 expression, collected clinicopathological and survival data, and explored the association between the LAPTM4B-35 protein expression and clinicopathological factors. Consistently, our study found that the LAPTM4B-35 was overexpressed in NSCLC compared to the normal tissues (Figure 1). We further investigated the associations of elevated LAPTM4B-35 protein expression and clinicopathological characters in NSCLC. As the Table 1 showed, the higher LAPTM4B-35 protein expression correlated with aggressive features (including poor histopathologic differentiation, lymph node metastasis and advanced clinical stage.) and tumor recurrence.

Recently, the prognostic significance of LAPTM4B-35 had been confirmed in several solid tumors [13, 22, 23]. In the present study, our data also identified the elevated LAPTM4B-35 protein level (HR = 2.879, 95% CI, 1.621-4.318; *p* = 0.018) (Table 3) was an independent prognostic factors for OS. And the 5-year median OS and PFS for NSCLC patients were significantly worse in the high LAPTM4B-35 protein expression group compared to low expression group (OS: *p* = 0.034; Figure 2A; PFS: *p* = 0.004; Figure 2B) (Figure 2).

The potential limitations including: the relative small size population limited the degree of evidence. Accordingly, larger and muti-center clinical study needs to be conducted to validate our report. Besides, our results and the previous studies all found the LAPTM4B-35 was associated with tumorigenesis, progression and aggressive clinicopathological features, and the potential molecular mechanisms for these processes need to be elucidate.

## CONCLUSIONS

Our study identified that the LAPTM4B-35 was elevated in NSCLC, the elevated LAPTM4B-35 expression was associated with aggressive clinicopathological features and poor prognosis, suggesting that LAPTM4B-35 protein could be applied in predicting patient's prognosis.

## MATERIALS AND METHODS

Patients who were confirmed NSCLC histologically from 2007 to 2011 were enrolled in this study. The inclusion criteria included: (a) received radical surgery; (b) had the complete clinical and follow-up informations. The exclusion criteria included: (a) distant metastasis was found before or during the operation; (b) patients who received treatment prior to radical surgery. Base on the above criteria, total of 107 patients were enrolled in this study. Written informed consents were obtained from all participants according to the Helsinki Declaration, and this study protocol was approved by the Ethics Committee of our hospital, Tianjin, China.

The clinical information such as age, gender, tumor size, histology, TNM stage, clinical stage and survival data were collected. The follow-up data was obtained from outpatient visit or telephone follow-up. The OS was defined as time from the day of diagnosis to the day of last visit or death. The median follow-up time was 64.5 months.

### RNA preparation, quantitative real-time PCR

Total RNA was extracted using Trizol reagent (Invitrogen). Quantitative real-time reverse transcription-polymerase chain reaction (qRT-PCR) was performed using SYBR Green polymerase chain reaction master mix according to the manufacturer's instructions (Takara). The primers were as follows:

LAPTM4B-35: forward 5′- GCCCGGAGCGATGAAGATG-3′, reverse 5′-CAACAGTACCACAGCATTGATGA-3′; D-glyceraldehyde-3-phosphate dehydrogenase (GAPDH): forward 5′-CCATCAATGACCCCTTCATTG-3′, reverse: 5′-GACGGTGCCATGGAATTT-3′. The cycling program was: denaturation at 95°C for 30 seconds, followed by 38 cycles of denaturing at 95°C for 5 seconds, annealing at 65°C for 30 seconds. The results of qRT-PCR were analyzed by using the 2^-ΔΔCt^method. GAPDH was used as an internal control.

### Western blot

Western blotting was conducted as previously described [14, 15]. In brief, tumor tissues were lysed with NP40 lysis buffer. Equal amounts of proteins were separated by SDS-PAGE, then the proteins were transferred to polyvinylidene fluoride (PVDF) membranes, blocked with 5% nonfat milk, and incubated with GAPDH and anti-LAPTM4B-35 polyclonal antibody (dilution, 1μg/ml; Abcam, Cambridge, UK.) at 4°C overnight, and then probed with secondary antibody at room temperature for 1 hour. Signals were detected using enhanced chemiluminescence detection system (Pierce, Rockford, IL, USA). The intensity of hybridization signals was quantified using Image analysis program. The protein levels were normalized to those of GAPDH. The LAPTM4B-35 protein levels were divided into two groups based on the median expression level.

### Statistical analysis

Continuous variables were described using mean ±standard deviation; the categorical variables were analyzed by a chi-squared test. The Kaplan-Meier method and the Multivariate analyses were conducted to identify significant independent factors for the prognosis. The statistical analyses were performed using SPSS version 18.0 (SPSS, Chicago, IL, United States). Significance was defined as *p*-Values (two sides) < 0.05.
